# A Network Pharmacology Approach to Explore the Mechanisms of Shugan Jianpi Formula in Liver Fibrosis

**DOI:** 10.1155/2020/4780383

**Published:** 2020-06-12

**Authors:** Chang Fan, Fu Rong Wu, Jia Fu Zhang, Hui Jiang

**Affiliations:** ^1^Experimental Center of Clinical Research, The First Affiliated Hospital of Anhui University of Chinese Medicine, Hefei 230031, Anhui, China; ^2^Department of Pharmacy, The First Affiliated Hospital of University of Science and Technology of China, Hefei 230001, Anhui, China; ^3^Anhui Province Key Laboratory of Chinese Medicinal Formula, Hefei 230031, Anhui, China

## Abstract

**Purpose:**

We explored the mechanism of Shugan Jianpi Formula (SGJPF) and its effective components for the treatment of liver fibrosis (LF).

**Materials and Methods:**

We collected the active ingredients in SGJPF through the Traditional Chinese Medicine Systems Pharmacology Database and Analysis Platform and screened the effective components by absorption, distribution, metabolism, and excretion. Herb-associated target proteins were predicted and screened based on the Bioinformatics Analysis Tool for Molecular Mechanism of Traditional Chinese Medicine and Search Tool for Interactions of Chemicals databases. LF-associated target proteins were predicted and screened based on the Online Mendelian Inheritance in Man® Database and Comparative Toxicogenomics Database. Common genes with LF and herbs were selected, and Cytoscape 3.5.1 software was used to construct an herb pathway and component-LF common target network. The Search Tool for the Retrieval of Interacting Genes/Proteins was used to build a protein-protein interaction, and quantitative PCR was used to verify the related target genes. Finally, clusterProfiler was applied for the analysis of Gene Ontology terms and Kyoto Encyclopedia of Genes and Genomes pathways.

**Results:**

The pharmacological network contained 252 active compounds (e.g., Astragaloside A, saikosaponin, linoleic acid, and Poria acid A), 84 common target genes, and 94 significant signaling pathways. Among them, interleukin 6 (IL-6), tumor protein 53 p53 (TP53), prostaglandin-endoperoxide synthase 2 (PTGS2), AKT1, IL-1*β*, and the nucleotide-binding and oligomerization domain-like receptor and Janus kinase-signal transducer and activator of transcription signaling pathways were selected as the critical target gene and critical signal pathway, respectively.

**Conclusion:**

The mechanisms of SGJPF in protecting against LF include the regulation of multiple targets such as IL-6, TP53, PTGS2, and AKT1. These target proteins affect LF through various signal transduction pathways.

## 1. Introduction

Liver fibrosis (LF) is a pathological reaction of the liver to chronic liver injury [1]. Liver injury leads to activation of hepatic stellate cells (HSCs) and an increase in extracellular matrix (ECM) synthesis and secretion. A large amount of ECM causes insufficient blood supply to the liver, and some HSCs are transformed into proliferative and contractile myofibroblasts, followed by the development of LF [2]. Without proper intervention, there is a risk that LF will further develop into cirrhosis or even liver cancer. Although LF is reversible, there is no specific drug for the treatment of hepatic fibrosis. Therefore, it is necessary to identify effective clinical treatments for LF.

Traditional Chinese Medicine (TCM) has received an increasing amount of attention due to its notable effectiveness and low side effects. TCM also has special advantages in antiliver fibrosis as it is characterized by holistic concepts and TCM syndrome differentiation and treatment [3]. Shugan Jianpi Formula (SGJPF), a classical TCM formula, consists of Baishao (BS, Paeoniae Radix Alba), Baizhu (BZ, Atractylodis Macrocephalae Rhizoma), Banlan Gen (BLG, Isatidis Radix), Chaihu (CH, Bupleuri Radix), Fuling (FL, Poria), Gancao (GC, Glycyrrhizae Radix et Rhizoma), Huangqi (HQ, Astragali Radix), Yiyi Ren (YYR, Coicis Semen), Yinchen (YC, Artemisiae Scopariae Herba), Zelan (ZeL, Lycopus lucidus Turcz), and Zhuling (ZL, Polyporus) and has been widely used to clinically treat liver diseases. Furthermore, SGJPF can reduce the levels of inflammatory factors, inhibit hepatic inflammatory injury, improve the degree of hepatocyte infiltration, and prevent liver-related histopathological changes; it may also exert antifibrosis effects by directly inhibiting HSC activation [4]. Due to the complexity of Chinese herbal formula, it is difficult to identify the effective mechanism of SGJPF with multicomponents, multitargets, and multieffects relying on the traditional pharmacological evaluation.

With the development of system biology and bioinformatics, analysis based on network pharmacology provides new methods that reveal the complex mechanisms of TCM. The methodologies of network pharmacology highlight the paradigm shift from “one drug, one target” to “multicomponent therapeutics, biological network” [5]. TCM has the advantages of multiple components and targets, which correspond to the methodologies of network pharmacology [6]. Thus, network pharmacology is desirable for exploring the mechanisms of TCM.

**Figure 1:**
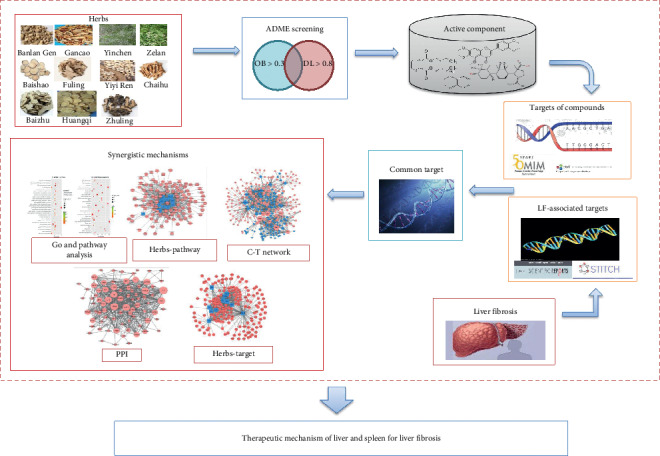
The reconstruction pipeline of the network pharmacology approach to explore the mechanisms of SGJPF in liver fibrosis.

In this study, to reveal the mechanism of SGJPF in the treatment of LF, we collected information on targets of active ingredients in SGJPF and targets of LF from several databases and performed network construction and topological structural analysis. The results of this study reveal the underlying synergistic mechanisms of SGJPF in treating LF (Figure 1).

## 2. Materials and Methods

### 2.1. Building Database of Ingredients

All data on the chemical ingredients of 11 herbs (Baishao, Baizhu, Banlan Gen, Chaihu, Fuling, Gancao, Huangqi, Yiyi Ren, Yinchen, Zelan, and Zhuling) were derived from the TCM Systems Pharmacology Database and Analysis Platform (TCMSP). TCMSP provides users with a platform for the analysis of TCM components, including the identification of active components [7].

### 2.2. Screening of Active Ingredients

In modern drug discovery, early assessment of absorption, distribution, metabolism, and excretion (ADME) screening has become an essential process. The proper use of ADME results can give preference to those drug candidates that are more likely to have good pharmacokinetic properties and minimize potential drug-drug interactions. In the present work, two ADME-related models, oral bioavailability (OB) and drug-like (DL), were employed to screen the active components from SGJPF. DL helps to describe the pharmacokinetic and pharmaceutical properties of compounds, such as solubility and chemical stability [8]. Usually, the selection criterion for “DL” compounds in TCM is 0.18. OB represents the relative amount of an oral drug that is absorbed into the blood [9]. Because low OB is the primary reason for the development of TCMs into therapeutic drugs, it is vital to conduct OB screening. Based on the literature and suggestions in TCMSP, we selected OB > 30% and DL > 0.18 as the screening threshold. The ingredients that meet the above criteria are screened out for further analysis. The network between herbs and active ingredients was visualized by Cytoscape 3.5.1 [10].

### 2.3. LF-Associated Target Prediction

Different genes associated with LF were collected from Online Mendelian Inheritance in Man (OMIM) (http://www.omim.org/) with keywords of “liver fibrosis” or “hepatic fibrosis” and the Comparative Toxicogenomics Database (CTD) (https://www.ctdbase.org) [11] with the keyword of “fibrosis.” The two databases manually curate information about gene–disease relationships.

### 2.4. Herb-Associated Target Prediction

Two databases were combined to predict relevant targets of active ingredients in SGJPF comprehensively. The Bioinformatics Analysis Tool for Molecular mechANism of TCM (BATMAN-TCM) (http://bionet.ncpsb.org/batman-tcm) ranks potential drug-target interactions based on their similarity to known drug-target interactions [12]. The Search Tool for Interactions of Chemicals (STITCH) database (http://stitch.embl.de/) integrates many sources of experimental and manually curated evidence with text-mining information and interaction predictions [13]. First, the active constituents were entered into BATMAN-TCM and STITCH databases. Then, duplications and unified names were removed from the targets obtained from the aforementioned two tools.

### 2.5. Herb-Pathway Network Construction

To analyze the main function of herb-target genes, Kyoto Encyclopedia of Genes and Genomes (KEGG) pathway analysis was performed using clusterProfiler with each herb's targets. Of note, a *P* value was implemented to determine the statistical significance of the modules. KEGG pathways with *P* < 0.05 are significant signaling pathways. Cytoscape 3.5.1 [10], an open-source software platform for visualizing complex networks, was employed to visualize the network.

### 2.6. Component-LF Common Target Network

Integrated targets were matched with the LF-associated target, and the intersecting genes were preserved for further analysis. A component-target network was established to identify the key target. Then, Cytoscape 3.5.1 was used to visualize the network [10]. Simultaneously, all node degrees of the component-target (C-T) network were calculated.

### 2.7. Protein-Protein Interaction Network Construction and Module Analysis

The Search Tool for the Retrieval of Interacting Genes/Proteins (STRING) (https://string-db.org/) is a database that provides comprehensive information about interactions between proteins, including prediction and experimental interaction data [14]. In our study, the STRING tool was used to generate protein-protein interactions (PPIs) among the target, based on interactions with a combined score ≥0.4. Cytoscape 3.5.1 was used to visualize the network [10].

### 2.8. Gene Ontology and Pathway Functional Enrichment Analysis

To analyze the main function of the target genes, KEGG pathway analysis was performed using clusterProfiler with each herb's targets. Of note, a *P* value was implemented to determine the statistical significance of the modules. KEGG pathways with *P* < 0.05 are significant signaling pathways. Cytoscape 3.5.1 was used to visualize the network [10].

### 2.9. Quantitative PCR Verifies the Effect of SCJPF on Gene Expression

An animal model of liver fibrosis was established by a single subcutaneous injection of 0.05 mL/10 g with 20% carbon tetrachloride (CCl4) into the back skin of mice, twice a week for 12 weeks. From the day after injection with 20% CCl4, SGJPF (7.80 g/kg) was administered by gavage once a day for 12 weeks. Then, total RNA from the liver was extracted using the EZ-10 Total RNA Mini-Prep Kit (B618583; Sangon Biotech Co., Ltd., Shanghai, China) and first-strand cDNA was reverse-transcribed and synthesized using the ABscript II cDNA First-Strand Synthesis Kit (RK20400; ABclonal Technology, Woburn, MA, USA). The reaction system was established by TB Green™ Premix Ex Taq™ II (RR820A; TAKARA, Tokyo, Japan), and quantitative PCR (qPCR) was performed using the Bio-Rad iQ5 Real-Time PCR System (IQ5; Bio-Rad, Hercules, CA, USA). The primers are shown in Table 1. The relative expression of each target gene was quantified and normalized by 2^−ΔΔCT^.

### 2.10. Statistical Analysis

SPSS17.0 software was used for data analysis and processing, and the results are expressed as mean ± standard deviation (SD) $\left(\bar{x}\pm s\right)$. One-way analysis of variance (ANOVA) designed by randomized block design was used to analyze the differences in multiple groups. The result was considered statistically significant when *P* < 0.05.

## 3. Results

### 3.1. Component Intersection in SGJPF

**Figure 2:**
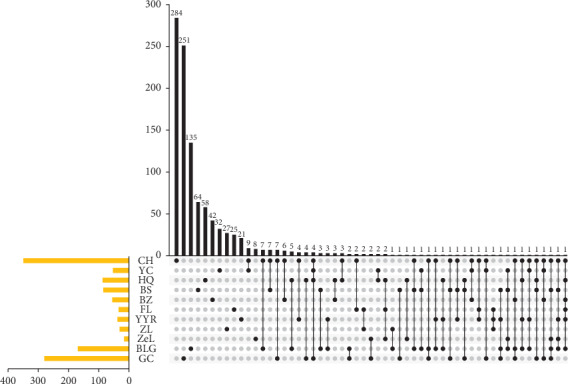
Upset chart showing the overlapping ingredients in each herb. The identified ingredients shared by different herbs are shown in the top bar chart. The specific names of each herb are indicated by the solid points below the bar chart.

In total, 11 types of herbs in the SGJPF contained 1048 compounds; detailed information about all compounds can be visualized in the Upset plot, which demonstrates the quantitative results of multiple interactive sets more effectively than the traditional Venn diagram [15]. As shown in Figure 2, it is noteworthy that >10% of compounds were found in two or more herbs, and different combinations of these herbs may provide the largest specific effect. For example, palmitic acid was in six types of herbs including Chaihu, Huangqi, Baizhu, Fuling, Yiyi Ren, and Banlan Gen. However, some compounds were unique to one herb. For instance, 284 components were only found in Chaihu, and 135 compounds were only found in Gancao.

### 3.2. Active Components of SGJPF

**Figure 3:**
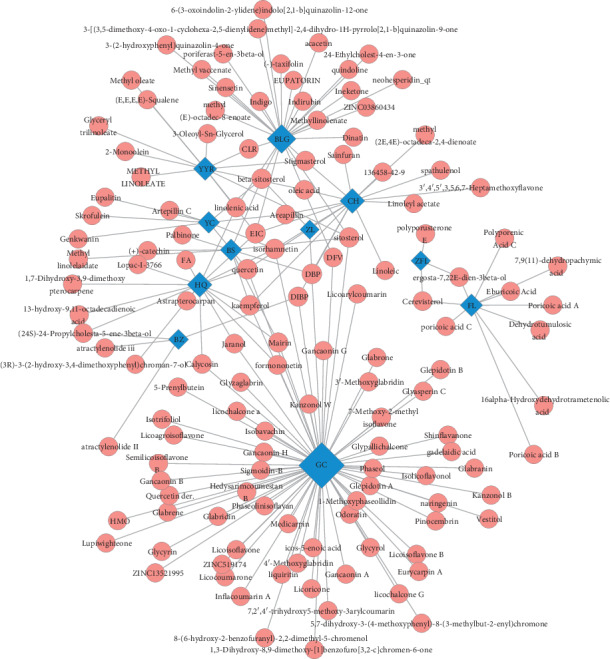
Herb-active compounds network. Blue squares represent 11 herbs in SGJPF. Red diamonds represent active compounds in herbs. Edges represent interaction between compounds and herbs.

According to the screening threshold of OB > 30% and DL > 0.18, 252 active compounds from all compounds were selected as the active components of SGJPF including astragaloside A, saikosaponin, linoleic acid, and Poria acid A (Figure 3).

### 3.3. Herb-Pathway Network

**Figure 4:**
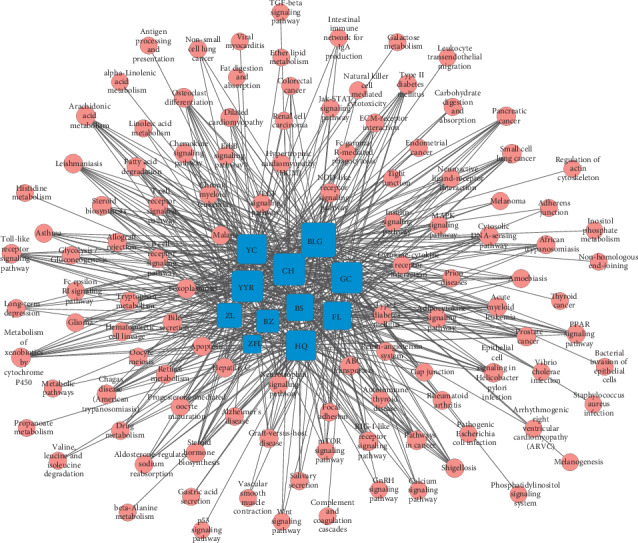
Herb-pathway network. Blue squares represent 11 herbs in SGJPF. Red circles represent enriched reactome pathways.

To explore the biological functions of herb-target genes, the KEGG pathway was analyzed, and then the herb-pathway network was reconstructed using Cytoscape 3.5.1. As shown in Figure 4, the herb-pathway network was composed of 11 herbs nodes, 109 pathways nodes, and 603 edges. In this research, 109 pathways were retrieved based on 1136 target genes predicted by herbs. These findings indicated that SGJPF inhibits LF through multiple pathways, which is consistent with the characteristics of TCM.

### 3.4. Common Target Prediction

TCM exerts its pharmacological action through the interaction of various components and targets. Therefore, in addition to the predictive component, studying the target is also necessary [16]. However, searching for targets through the literature is time consuming and labor intensive. In this work, predictive models including BATMAN-TCM and STITCH were used to predict 5544 targets, which interacted with the active ingredients. In addition, the OMIM database and CTD were also used to predict 983 targets relating to LF. After merging these genes, 170 common target genes were acquired.

### 3.5. Compound-LF Common Target Network

**Figure 5:**
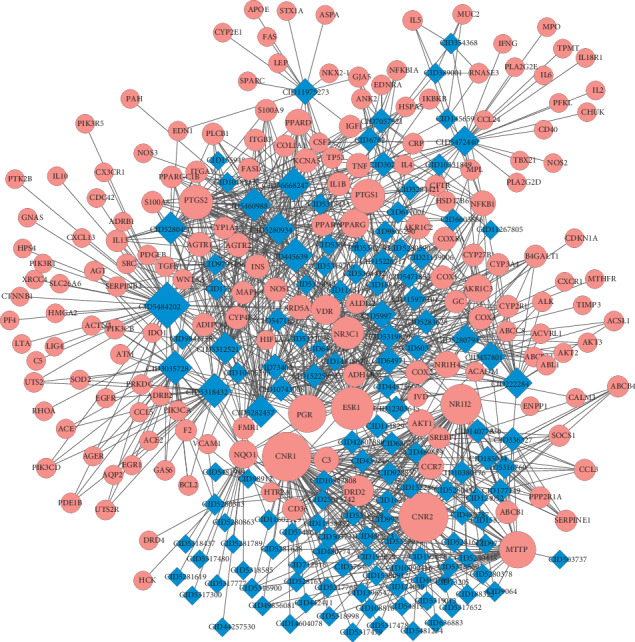
Ingredient-target network. Red circles represent active compounds in SGJPF. The blue rhombus represents common targets between compounds' targets from SGJPF and LF significant targets. Edges represent interaction between compounds and targets.

To reflect the intrinsic connection among compounds, diseases, and target genes, a network among compounds and common target genes was reconstructed and visualized. As shown in Figure 5, the compound-target network comprised 301 nodes including 131 active ingredient nodes, 170 target genes nodes, and 1181 edges. According to Figure 5, we also found that rather than a one-to-one relationship between compounds and genes, a compound could correspond with multiple target genes, just as a gene can correspond with multiple compounds.

### 3.6. PPI Network

**Figure 6:**
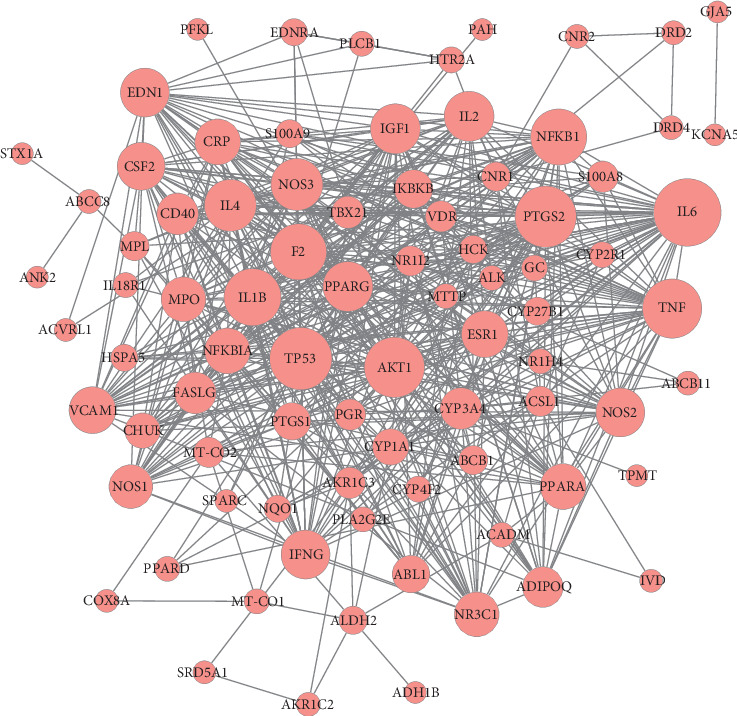
PPI network of common targets. Red circles represent common targets between ingredient targets from SGJPF and LF significant targets.

**Figure 7:**
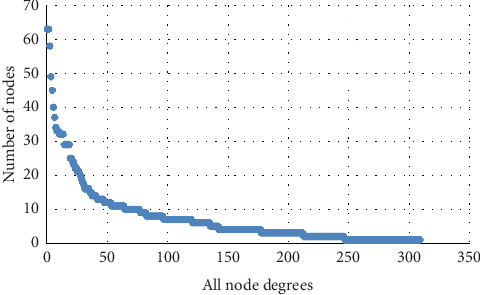
All-node degree analysis reveals specific targets of PPI network.

After removing isolated genes, the PPI network was delineated using the STRING database. As shown in Figure 6, the PPI network contained 84 nodes and 570 edges. Genes with a high degree of connectivity in the PPI network may be potential targets for diseases [17]. Genes were ranked by degree as shown in Table 2 and Figure 7, suggesting that those with the highest degree may play crucial roles in the origin and development of LF.

### 3.7. Biological Function and Pathway Enrichment Analysis of Core Targets

**Figure 8:**
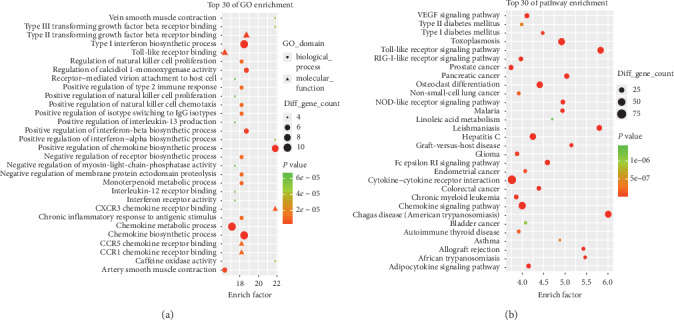
GO and pathway analysis of the targets of common targets. (a) The top 30 significant GO terms. (b) The top 30 signaling pathways. The different colors from green to red represent the *P* value. The different sizes of the shapes represent the gene count number. The larger the proportion is, the larger the dot; the redder the dot color, the more significant the *P* value.

To explore the biological functions of core targets, Gene Ontology (GO) and KEGG pathways were analyzed. GO analysis revealed that there were 3626 GO terms. Among them, biological processes included positive regulation of type 2 immune response, chronic inflammatory response to antigenic stimulus, negative regulation of receptor biosynthetic process, and positive regulation of natural killer cell chemotaxis. The molecular functions mainly included the chemokine metabolic process, type II transforming growth factor beta receptor binding, interleukin-12 (IL-12) receptor binding, and toll-like receptor (TLR) binding. The top 30 significant GO terms are shown in Figure 8(a). KEGG pathway analysis indicated that 94 enrichment pathways were screened by *P* < 0.05, which were mainly related to the TLR and nucleotide-binding oligomerization domain (NOD)-like receptor signaling pathways. The top 30 pathways are shown in Figure 8(b).

### 3.8. Effect of SGJPF on the mRNA Expression of IL-6, Tumor Protein p53, AKT1, and IL-1*β*

**Figure 9:**
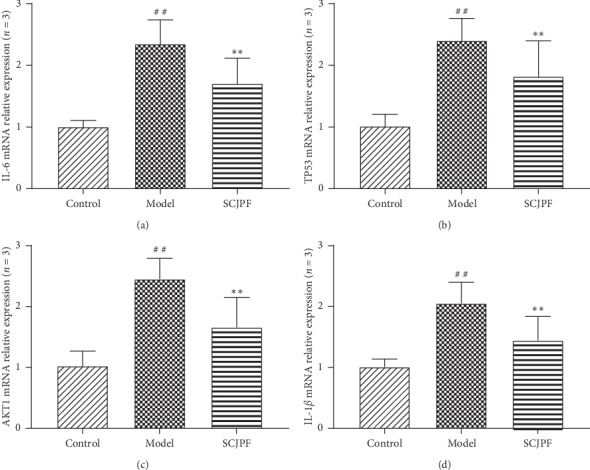
Change in IL6, TP53, AKT1, and IL-1*β* mRNA expression was observed by qPCR. (a) Change in IL-6 mRNA expression was observed by qPCR. (b) Change in TP53 mRNA expression was observed by qPCR. (c) Change in AKT1 mRNA expression was observed by qPCR. (d) Change in IL-1*β* mRNA expression was observed by qPCR. ^##^*P* < 0.01 compared with the control group, ^*∗∗*^*P* < 0.01 compared with the model group.

We chose four target genes with higher degrees to verify the influence of SGJPF. As shown in Figure 9, compared with the control group, the expression levels of IL-6, tumor protein p53 (TP53), AKT1, and IL-1*β* in the model group were significantly increased, while the expression levels of IL-6, TP53, AKT1, and IL-1*β* in the SCJPF group were significantly lower than those in the model group.

## 4. Discussion

LF is considered the common pathological process of all chronic liver diseases, with complex formation processes and mechanisms, many influencing factors, and great individual differences. Therefore, the clinical treatment of LF still faces great challenges [18]. In view of the pathogenesis of LF, inhibition of HSC proliferation, anti-inflammation, and protection of hepatocytes are key to the treatment of LF. It has been proven that SGJPF has curative effects on LF induced by CCl4 in rats [19].

In light of the complexity of the active ingredients in SGJPF and the diversity of potential regulatory targets in the human body, we analyzed the main components of SGJPF by network pharmacology. The holistic and systematic nature of network pharmacology coincides with the holistic view of TCM and syndrome differentiation. It can use many existing databases and pathway and network analyses to explore the molecular mechanism of TCM for the treatment of diseases [20]. Then, we constructed the SGJPF-compound target-LF network, and the potential antihepatic fibrosis mechanism of SGJPF was predicted. Network pharmacological analysis of SGJPF identified 11 herbs, 252 active compounds, 84 common target genes, and 94 significant signal pathways regulated by target genes.

Astragaloside, the effective component of *Astragalus* membranaceus [21], attenuates LF by inhibiting the expression of proteinase-activated receptor 2 signal transduction, and its effect is greatly enhanced in diabetic animals [22]. Astragaloside also inhibits oxidative stress and activation of the p38/MAPK to reduce HSC activation [23]. Saikosaponins and linoleic acid in Bupleurum have protective effects on the liver, and saikosaponins have good antifibrosis effects, which are related to increasing superoxide dismutase activity, reducing the production of oxygen free radicals, and inhibiting activation of the transforming growth factor beta 1 signal pathway [24, 25]. Linoleic acid can inhibit the development of LF by inhibiting the activation or proliferation of HSCs [26]. Poria acid *A* is an active ingredient present in sputum and a well-known medicinal bacterium with various biological functions such as anti-inflammatory and antitumor effects. Studies have shown that Poria acid *A* has inhibitory effects on the formation of cell fibrosis [27].

According to the common target gene degree, this study predicted that SGJPF treats LF mainly through targeting IL-6, TP53, PTGS2, and AKT1, suggesting that SGJPF has multicomponents and multitargets characteristics for the treatment of LF [28]. The abnormal dynamic balance of cytokine networks is an important pathogenesis in the development and progression of LF. IL-6, a type of *T* cell and macrophage, is an important central gene [29]. Cytokines are involved in a variety of biological activities. IL-6 not only promotes the occurrence of LF by mediating inflammation but also participates in the formation of LF by promoting the synthesis of ECM. Some studies have shown that the level of serum IL-6 increases with the aggravation of LF, which is positively correlated with the levels of serum hyaluronic acid, type III procollagen, type IV collagen, and stage of LF, indicating that the increase of IL-6 level can lead to the formation and aggravation of LF [30]. TP53 is a transcription factor that is widely expressed in embryos and tumor tissues. Studies have shown that TP53 plays a regulatory role in muscle cell differentiation, suggesting that the recognition of new target molecules may bring new implications for the treatment of LF. Prostaglandin-endoperoxide synthase 2 gene is generally highly expressed in tissues with inflammation or tumorigenesis, stimulated by certain cytokines or inflammatory factors, suggesting that it may play a key role in cell proliferation and differentiation such as promoting the proliferation and differentiation of HSCs [31]. AKT1 is a serine/threonine protein kinase. Serine is an important amino acid responsible for the synthesis of cell membrane, muscle tissue, and nerve cells. Threonine promotes the synthesis of phospholipids and plays an important role in protecting the cell membrane [32]. By autophosphorylation, AKT1 regulates key signaling pathways such as the Janus kinase-signal transducer and activator of transcription (JAK-STAT), apoptosis, mammalian target of rapamycin, and mitogen-activated protein kinase (MAPK) signaling pathways [33, 34], regulates apoptosis and related proteins, thus affecting the process of LF. IL-1*β*, a critical mediator of the inflammatory response, and can promote the proliferation and differentiation of HSCs in a dose-dependent manner, leading to the occurrence and development of LF [35, 36]. Through qPCR, we confirmed that SCJPF can downregulate the expression of IL-6, TP53, AKT1, and IL-1*β* in the pathogenesis of LF to play a role in the treatment of LF.

The results of KEGG metabolic pathway enrichment analysis showed that the TLR, NOD-like receptor, and JAK-STAT signaling pathways were mainly involved in the treatment of LF with SGJPF. The TLR signaling pathway is complex and has a wide range of effects. Its effect on LF is mainly through TLR4, TLR9, and its signal transduction pathway, especially the signal pathway of myeloid differentiation factor 88-nuclear factor kappa B (NF-*κ*B) [37, 38]. After recognizing its ligand, TLR initiates the downstream signal pathway, produces a large number of inflammatory cytokines, and causes an inflammatory response, which in turn promotes the activation of HSCs [39]. NOD-like receptors, a part of the innate immune response, participates in the disease process of LF mainly through the NF-*κ*B pathway [40]. Activation of the NF-*κ*B pathway promotes the release of proinflammatory cytokines, during the occurrence of liver inflammation. NLR Family Pyrin Domain Containing 3 (NLRP3) inflammatory bodies are activated, and the expression of NLRP3, apoptosis-associated speck-like protein containing a CARD, and caspase-1 in the liver increases, which induces pyroptosis, further expanding the inflammatory response and promoting the occurrence and development of LF [41]. The JAK/STAT signaling pathway is widely involved in the processes of cell proliferation, differentiation, apoptosis, and inflammation and is an important signal transduction pathway for many cytokines [42, 43]. It also plays an important role in the formation of LF. Various cytokines such as leptin, interferon gamma, and IL-4 activate the intracellular JAK/STAT pathway through specific receptors on the HSC membrane and transduction signals into the nucleus, promoting the transcription and expression of related target genes [44].

There are still some limitations in using network pharmacology to predict the effective components and potential mechanism of SGJPF. First, when predicting the potential active components of SGJPF, OB and DL in TCM powder were used as screening indexes. However, compared with the data from other databases (e.g., BATMAN-TCM and TCMID), the screening indicators are different. After consideration, we only retained the data from TCMSP, but did not rule out the existence of other active ingredients for the treatment of LF. Second, the data obtained in this study were based on previous experimental results and computer simulation predictions. Although we obtained some action targets and the pathways in which these targets are located to provide a direction for follow-up research, the more exact therapeutic mechanism of SGJPF on LF was not reflected in this study. Therefore, this still needs to be studied or verified in detail in follow-up animal and human experiments to further implement the development of new drugs and improve clinical medication thinking. Third, during the treatment of LF with SGJPF, the drug components actually absorbed into the patient's body after oral administration are not necessarily the same as the active components selected; thus, the effect of drugs on the target may also be different, and the effects of other factors may have led to deviations in the results. Therefore, to verify the theoretical prediction, further experiments on the effective components are needed.

## 5. Conclusion

In conclusion, the mechanism of SGJPF in the treatment of LF involves a variety of active components, targets, and pathways. In this study, 252 active components, 84 potential targets, and 94 related signaling pathways in SGJPF were predicted. Among them, astragaloside *A*, saikosaponin, Poria acid *A*, and other components may act on IL-6, TP53, PTGS2, AKT1, and other targets, affecting TLR, NLR, and JAK/STAT, among other signaling pathways and participating in the inflammatory response, HSC proliferation and differentiation, apoptosis, pyroptosis, and other processes. This appears to be the mechanism underlying the therapeutic effect of SGJPF on LF, and it broadens the train of thought for follow-up pharmacological research.

## Figures and Tables

**Table 1:** Information of primers for RT-qPCR.

Targeted gene	Forward sequence and reverse sequence	Product length (bp)
IL-6	F: 5′-TTCACAAGTCGGAGGCTTA-3′	105
	R: 5′-CAAGTGCATCATCGTTGTTC-3′	

TP53	F: 5′-ACCTGAAGACCAAGAAGGG-3′	225
	R: 5′-GACCGGGAGGATTGTGT-3′	

AKT1	F: 5′-TAACGGACTTCGGGCTGT-3′	480
	R: 5′-TTCTCGTGGTCCTGGTTGT-3′	

IL-1*β*	F: 5′-AGTTGACGGACCCCAAA-3′	424
	R: 5′-TCTTGTTGATGTGCTGCTG-3′	

**Table 2:** Common target gene dimension ranking.

Genes	Degree
IL6	45
TP53	40
PTGS2	39
AKT1	38
TNF	37
IL1*β*	35
NFKB1	34
F2	34
IL4	30
NOS3	30
IL2	28
PPARG	28
IGF1	28
IFNG	27
NOS2	27
EDN1	27
CSF2	26
ESR1	25
VCAM1	25
NFKBIA	24
CRP	24
PPARA	24
MPO	23
NR3C1	23
NOS1	22
CYP3A4	20
FASLG	20
CD40	20
ADIPOQ	19
IKBKB	17
ABL1	16
PTGS1	16
CYP1A1	15
CHUK	14
TBX21	11
S100A8	10
AKR1C3	10
PGR	10
VDR	10
NR1I2	9
ACSL1	9
CNR1	8
MT-CO2	9
ABCB1	8
MPL	8
ALDH2	7
CYP27B1	7
HCK	7
S100A9	7
EDNRA	6
NQO1	6
HSPA5	6
CYP4F2	5
HTR2A	5
GC	5
CYP2R1	5
MT-CO1	4
PLCB1	4
IL18R1	4
ACADM	4
PPARD	4
MTTP	4
NR1H4	4
PLA2G2E	4
ALK	4
DRD2	3
DRD4	3
ABCB11	3
AKR1C2	3
CNR2	3
ABCC8	3
SRD5A1	2
SPARC	2
COX8A	2
IVD	2
ACVRL1	2
PFKL	1
STX1A	1
KCNA5	1
TPMT	1
ANK2	1
ADH1B	1
PAH	1
GJA5	1

## Data Availability

The datasets used and/or analyzed during the present study are available from the corresponding author upon reasonable request.
